# *TP53* and *MDM2* polymorphisms and the risk of endometrial cancer in postmenopausal women

**DOI:** 10.1007/s12032-014-0286-z

**Published:** 2014-10-15

**Authors:** Agnieszka Zając, Beata Smolarz, Grzegorz Stachowiak, Jacek R. Wilczyński

**Affiliations:** 1 Department of Gynecology and Gynecologic Oncology, Polish Mother’s Memorial Hospital, Research Institute, 281/289 Rzgowska St., 93-338 Lodz, Poland; 2 Laboratory of Molecular Genetics, Department of Pathology, Polish Mother’s Memorial Hospital, Research Institute, 281/289 Rzgowska St., 93-338 Lodz, Poland

**Keywords:** *TP53*, *MDM2*, Genetic polymorphisms, Endometrial carcinoma, Menopause

## Abstract

The aim of the study was to determine an association of *TP53* codon 72 (Arg72Pro, G>C transversion, rs1042522) and *MDM2* SNP309 (T>G change, rs2279744) polymorphisms in endometrial cancer (EC) of postmenopausal women, regarding grading and staging of EC. In the study, endometrial samples from 202 postmenopausal female patients (the study group, *n* = 152, was women with EC; the control group, *n* = 50, cancer-free patients) were taken for the evaluation of two gene polymorphisms: *TP53* codon 72 and *MDM2* SNP309, respectively. Genotypic analyses were performed using the PCR–RFLP technique. There were significant differences in the frequency of *TP53* and *MDM2* genotypes in EC patients—increased EC occurrence was observed with the presence of *MDM2* G/G and *TP53* Arg/Arg genotypes, while allele Pro of *TP53* decreased cancer risk. Analysis of combined *MDM2/TP53* polymorphisms revealed that T/T-Pro/Arg genotype decreased EC risk, whereas G/G-Arg/Arg genotype increased it. Association of these genetic polymorphisms with histological grading showed increased *MDM2* G/G homozygote and *TP53* Arg/Arg homozygote frequencies in grading 2 as well as allele G overrepresentation in G1 and G3 EC patients. Finally, with clinical FIGO staging under evaluation, an increase in *MDM2* G/G and TP53 Arg/Arg homozygote frequencies in staging I and *TP53* Arg/Arg homozygote frequencies in staging II were observed. Co-occurrence of some *MDM2* SNP309 and *TP53* codon 72 polymorphisms seems to influence EC risk, involving grading and staging of this neoplasm at the same time.

## Introduction

Genetic base of endometrial cancer (EC), the most frequent neoplasm of sex organs in postmenopausal women, is not fully understood. Among numerous genes postulated to be involved in EC oncogenesis, at least two seem to play here an important role. These genes—*MDM2* and *TP53*—are taking part in cell-cycle regulation. The first one—*MDM2*—belongs to proto-oncogenes, or proliferation genes responsible for cell growth and differentiation. The second one—*TP53*—is a suppressor gene preventing genetic anomalies transmission to daughter cells. *TP53* in the moment of DNA strand damage inhibits cells in G1 phase and runs repair mechanisms or apoptosis when damage is too much [1, 2].

From oncogenic point of view extremely important appears to be that action of these two genes is interrelated: Protein product of *TP53* gene—p53 protein—has the ability to activate (a number of genes including) *MDM2* gene, resulting in an increased production of mdm2 protein, which in turn inhibits p53 protein activity and by reducing its suppressor properties may initiate the process of carcinogenesis, including EC as well [3]. Any disruption in *MDM2/TP53* genes may underlie EC development. Unfortunately, data on the occurrence of specific *MDM2/TP53* mutations or polymorphisms in EC are scant, mostly analyzing each gene separately, not together [4, 5].

The aim of this present work was to change the existing situation and to determine the potential relationship between *TP53* codon 72 and *MDM2* SNP309 polymorphisms in EC of postmenopausal women, regarding histological malignancy (grading) and clinical staging of this neoplasm.

## Materials and methods

### Endometrial cancer patients

A total of 152 patients with histologically proven diagnosis of endometrial cancer were included in the study (Table 1). Paraffin-embedded tumor tissues were obtained from postmenopausal women (aged 60.90 ± 8.96) with endometrial carcinoma treated in Polish Mothers Memorial Hospital, Research Institute (PMMH,RI, Lodz, Poland) between 2004 and 2009 years. All tumors were staged according to the criteria of the International Federation of Gynaecology and Obstetrics (FIGO). DNA from normal endometrial tissue obtained from non-cancer patients (*n* = 50, aged 53.06 ± 4.75) served as control. Normal endometrial specimens were obtained from patients who had undergone hysterectomy for intramural leiomyomas. The Local Ethic Committee approved the study, and each patient gave a written consent.

**Table 1:** Characteristics of the study population (*n* = 152)

Characteristics	Number of cases (%)
BMI (body mass index) (kg/m^2^)	
<18	0
18–25	21 (14 %)
26–29	60 (39 %)
>30	71 (47 %)
Number of birth	
0	48 (32 %)
1	104 (68 %)
>1	0
First menarche	
Before 11 years	6 (4 %)
12–13 years	65 (43 %)
14–15 years	71 (47 %)
After 16 years	10 (6 %)
FIGO grade	
G1	83 (54 %)
G2	34 (22 %)
G3	35 (23 %)
FIGO stage	
I	94 (62 %)
II	36 (24 %)
III	22 (14 %)
Use of hormone replacement therapy—HRT	
Yes	96 (63 %)
No	56 (37 %)
Endometrial ultrasound transvaginal—TVU	
>5 mm	115 (75 %)
Diabetes mellitus	28 (18 %)
Hypertension	80 (53 %)
Uterine bleeding	100 (65 %)

### DNA isolation

DNA for analysis was obtained from an archival pathological paraffin-embedded tumor and healthy endometrial samples, which were deparaffinized in xylene and rehydrated in ethanol and distilled water. For tissue deparaffinization, 1,200 μl xylene was added to tissue section and agitated for 5 min, then centrifuged at 12,000 rpm for 10 min. The supernatant was removed and fresh xylene was added, and this step was repeated five times followed by washing with 100 % ethanol for 10 min and centrifuging at 12,000 rpm for 10 min. Then, the tissue pellet was air-dried. 180 µl of DNA extraction buffer solution ATL (Qiagen GmbH, Hilden, Germany) was added to the deparaffinized tissue in a 1.5-ml microcentrifuge tube followed by the DNA extraction step. Genomic DNA was prepared from material by using the commercial QIAmp DNA Kit (Qiagen GmbH, Hilden, Germany) according to the manufacturer’s instruction.

### Determination of MDM2 genotype

Genotypic analysis of the *MDM2* SNP309 (rs2279744) polymorphism was determined by the PCR-based restriction fragment length polymorphism (PCR–RFLP) method. Genome region that includes studied polymorphism was amplified by PCR using primers 5′- CGCGGGAGTTCAGGGTAAAG-3′ and 5′-AGCTGGAGACAAGTCAGGACTTAAC-3′ [6, 7]. The PCR (total volume 25 μl) was performed with a mixture containing about 100 ng of DNA, 12.5 pmol of each primer, 0.2 mmol/l of dNTPs, 2 mmol/l of MgCl_2_ and 1 U of Taq DNA polymerase (TaKaRa, Japan). PCR conditions were as follows: initial denaturation step at 95 °C for 5 min, 35 cycles at 94 °C for 30 s and 30 s at the 62 °C annealing temperature, and at 72 °C for 30 s. The final extension step was performed at 72 °C for 5 min. The PCR was carried out in a PTC-100TM (MJ Research, INC, Waltham, MA, USA) thermal cycler. Following PCR, 20-ml aliquots were removed and subjected to restriction digestion with *MspA*1I (BioLabs, New England, Frankfurt am Main, Germany). The 237-bp amplified product was digested overnight with 1 U of *MspA*1I at 37 °C. The digested products were resolved on a 2 % agarose gel and stained with 0.5 μg/ml ethidium bromide. The wild-type allele *T* was identified by the presence of 237-bp band, while the mutant allele G was represented by 189- and 48-bp bands.

### Determination of p53 genotype

The detection of *p53* codon 72 (rs1042522) polymorphism was carried out using PCR–RFLP technique [8, 9]. A 309-bp fragment from exon 4 of p53 containing codon 72 *BstU*1 polymorphism site was amplified using the following exon 4 primers: forward primer 5′TTC ACC CAT CTA CAG TCC 3′ and reverse primer 5′CTC AGG GCA ACT GAC CGT 3′. The PCR was carried out in a PTC-100TM (MJ Research, INC, Waltham, MA, USA) thermal cycler. The 25 μl PCR mixture contained about 100 ng of DNA, 12.5 pmol of each primer, 2 μl dNTP (10 mM), 2 mmol/l of MgCl_2_ and 1 U of Taq DNA polymerase (TaKaRa, Japan). The PCR cycle conditions were 94 °C for 4 min, initial denaturation and 94 °C for 30 s, 62 °C for 30 s then 72 °C for 30 s, repeated for 35 cycles. The 309-bp amplified product was digested overnight with 1 U of *BstU*1 (BioLabs, New England, Frankfurt am Main, Germany) at 60 °C. After digestion, the fragments were electrophoresed on 2 % agarose gel and visualized by UV light after ethidium bromide staining. The Pro allele was 309 bp, while the Arg allele was restricted into two fragments of 175 and 134 bp.

### Statistical analysis

For each polymorphism, deviation of the genotype frequencies in the controls from those expected under Hardy–Weinberg equilibrium was assessed using the standard chi-squared test. Genotype frequencies in cases and controls were compared by chi-squared tests. The genotypic-specific risks were estimated as odds ratios (ORs) with associated 95 % intervals (CIs) by unconditional logistic regression. *P* values <0.05 were considered to be significant. Analyses were performed using STATISTICA 10 package (Statsoft, Tulsa, OK, USA).

## Results

All the recruited both endometrial cancer (*n* = 152) and control samples (*n* = 50) were successfully analyzed for the *TP53* and *MDM2* genotype. From the PCR analysis, all patients were classified into three genotypes of the *MDM2* and *TP53* gene: G/G, G/T and T/T and Pro/Pro, Pro/Arg and Arg/Arg, respectively.

It can be seen from Table 2 that there are significant differences in the frequency of *TP53* and *MDM2* genotypes (*p* < 0.05). An association was observed between endometrial carcinoma occurrence and the presence of G/G and Arg/Arg genotypes (Tables 3, 4, 5). Variant Pro allele of *TP53* decreased cancer risk (Table 3).

**Table 2:** Distribution of *TP53*-Pro/Arg and *MDM2*-G/T genotypes in patients with EC

	G/G	G/G (%)	T/G	T/G (%)	T/T	T/T (%)
Pro/Pro	37.00	0.37	10.00	0.33	16.00	0.66
Arg/Arg	31.00	0.31	12.00	0.4	7.00	0.29
Pro	104.00	0.53	28.00	0.46	33.00	0.68
Arg	92.00	0.46	32.00	0.53	15.00	0.31
chi^2^/*p*	14.56	**0.0002**	6.47	**0.02**	19.57	**0.00009**
Pro/Arg	30.00	0.30			1.00	0.04
Arg/Pro			8.00	0.26		

Bold values are statistically significant (*p* < 0.05)

**Table 3:** Distribution of genotypes and odds ratios (OR) of the *TP53*-Pro/Arg polymorphism in patients with *MDM2*-G/G homozygous variant and controls

	G/G	Controls	OR	X2.5..	X97.5	Z value	Pr…z..
Pro/Pro	37.00	22.00	0.77	0.39	1.55	−0.73	0.46
Pro/Arg	30.00	21.00	0.61	0.30	1.24	−1.37	0.16
Arg/Arg	31.00	7.00	2.84	1.20	7.54	2.26	**0.02**
Pro	104.00	65.00	0.35	0.13	0.83	−2.26	**0.02**
Arg	92.00	35.00	1.30	0.65	2.59	0.73	0.46

Bold values are statistically significant (*p* < 0.05)

**Table 4:** Distribution of genotypes and odds ratios (OR) of the *TP53*-Pro/Arg polymorphism in patients with *MDM2*-T/G heterozygous variant and controls

	T/G	Controls	OR	X2.5..	X97.5	Z value	Pr…z..
Arg/Arg	12.00	7.00	4.10	1.42	12.66	2.55	**0.01**
Pro/Pro	10.00	22.00	0.64	0.24	1.61	−0.94	0.34
Arg	32.00	35.00	1.57	0.62	4.14	0.94	0.34
Pro	28.00	65.00	0.24	0.08	0.70	−2.55	**0.01**

Bold values are statistically significant (*p* < 0.05)

**Table 5:** Distribution of genotypes and odds ratios (OR) of the *TP53*-Pro/Arg polymorphism in patients with *MDM2*-T/T homozygous variant and controls

	T/T	Controls	OR	X2.5..	X97.5	Z value	Pr…z..
Pro/Pro	16.00	22.00	2.55	0.94	7.31	1.80	0.07
Pro/Arg	1.00	21.00	0.06	0.00	0.32	−2.65	**0.008**
Arg/Arg	7.00	7.00	2.53	0.76	8.49	1.53	0.12
Pro	33.00	65.00	0.40	0.12	1.31	−1.53	0.12
Arg	15.00	35.00	0.39	0.14	1.06	−1.80	0.07

Bold value is statistically significant (*p* < 0.05)

We also analyzed combined genotype of all polymorphism pairs. The combined T/T-Pro/Arg genotype decreased the risk of endometrial cancer occurrence (Table 5). Moreover, the combined G/G-Arg/Arg genotype increased the risk of EC (Table 6).

**Table 6:** Distribution of *MDM2*-G/G and *TP53*-Arg/Arg genotypes and odds ratios (OR) in patients with EC and controls

	Patients	Controls	OR	X2.5..	X97.5	Z value	Pr…z..
G/G	98	14	4.79	2.39	10.06	4.30	**0.000016**
Arg/Arg	50	7	3.16	1.35	8.39	2.50	**0.01**

Bold values are statistically significant (*p* < 0.05)

The observed genotype frequency of *TP53* codon 72 (*p* > 0.05) as well as *MDM2* SNP309 in the controls group was in agreement with Hardy–Weinberg equilibrium (HWE) (*p* > 0.05). In case of both investigated genes, the distribution of the genotypes in the patients differed significantly from the one expected from the Hardy–Weinberg equilibrium (*p* < 0.05).

It is caused by the very low abundance of the *TP53* Pro/Pro genotype and *MDM2* T/T genotype in the examined Polish population.

Because we were interested in the association between the distribution of genotypes and frequencies of alleles of investigated genetic variability on the tumor grade evaluated according to FIGO criteria, these data were also analyzed. Histological grading was evaluated in all the cases (*n* = 152): grade 1 (G1)—83 cases, grade 2 (G2)—34 cases and grade 3 (G3)—35 cases (see Table 7). Some correlation was observed between the *TP53* Pro/Arg and *MDM2* G/T genotype and EC invasiveness. A strong increase was observed, regarding G/G homozygotes frequency and Arg/Arg homozygotes in G2 patients. That increase was statistically significant (*p* < 0.05). Moreover, EC patients in G1 and G2 had an overrepresentation of G alleles.

**Table 7:** Dependency of *MDM2*-G/G and *TP53*-Arg/Arg genotypes on the tumor grading in patients with EC

		Controls	OR	X2.5..	X97.5	Z value	Pr…z..
Grading G1							
G/G	44	14	5.19	2.35	12.00	3.98	**0.000069**
Arg/Arg	17	7	2.19	0.80	6.56	1.48	0.14
Grading G2							
G/G	23	14	2.82	1.17	7.11	2.26	**0.02**
Arg/Arg	19	7	4.68	1.74	13.84	2.95	**0.0031**
Grading G3							
G/G	14	14	4.99	1.68	16.14	2.81	**0.0049**
Arg/Arg	7	7	2.89	0.78	10.97	1.59	0.11

Bold values are statistically significant (*p* < 0.05)

Clinical FIGO staging was also related to *MDM2* G/T and the *TP53* Pro/Arg polymorphisms (Table 8). Staging was evaluated in all the cases (*n* = 152). An increase was observed, regarding G/G and Arg/Arg homozygotes frequency in staging I (SI) patients, according FIGO classification. That increase was statistically significant (*p* < 0.05). Moreover, in case of *TP53,* an increase was observed, regarding Arg/Arg homozygotes frequency in FIGO staging II (SII). That increase was also statistically significant (*p* < 0.05).

**Table 8:** Dependency of *MDM2*-G/G and *TP53*-Arg/Arg genotypes on the tumor staging in patients with EC

		Controls	OR	X2.5..	X97.5	Z value	Pr…z..
Staging I							
G/G	67	14	6.95	3.24	15.67	4.85	**0.0000012**
Arg/Arg	27	7	2.84	1.09	8.22	2.05	**0.04**
Staging II							
G/G	9	14	1.71	0.56	5.18	0.96	0.33
Arg/Arg	9	7	4.18	1.30	14.06	2.39	**0.017**
Staging III							
G/G	2	14	0.38	0.05	5.18	−1.16	0.25
Arg/Arg	5	7	3.18	0.79	12.67	1.66	0.10

Bold values are statistically significant (*p* < 0.05)

Our data did not demonstrate any statistically significant correlation between MDM2 and TP53 polymorphisms and the risk factors for endometrial cancer, such as body mass index, hormone replacement therapy, uterine bleeding, endometrial transvaginal ultrasound, diabetes and hypertension and women with endometrial cancer (data not shown).

## Discussion

The authors coping with genetic background of EC found that both *MDM2* and *TP53* polymorphisms are associated with the risk of this female neoplasm when evaluated separately.

In case of *MDM2*–EC relationship, an American publication by Walsh et al. [10] found that the presence of homozygous *MDM2* SNP309 G/G genotype is quite frequent—25 % in EC cases (11 % in controls), increasing the EC risk up to 2.76, having no association with EC grading and staging as well as patient’s age at diagnosis. Also, another American publication by Terry et al. [11], nested within two large case–control studies—WHS (Women’s Health Study) and NHS (Nurses’ Health Study) with a total number of 592 EC cases and 1.543 controls, reported increased EC risk (OR = 1.87) in women with G/G genotype of *MDM2* SNP309 polymorphism. In a Japanese report by Ueda et al. [12], both G/G genotype and G allele increased the risk of EC development, with OR = 1.91 and 1.20, respectively. In this study, homozygous G/G genotype was positively associated with postmenopausal status and EC type I alike, suggesting that this kind of *MDM2* SNP309 polymorphism could be a risk factor for EC type I in Japanese female population [12]. These results are in accordance with our previous reports, indicating that both G/G genotype and G allele are highly associated with increased EC risk in Polish women, OR—3.50 and 2.67, respectively. No relationship of this *MDM2* SNP309 polymorphism with EC grading was observed by us in this study [4].

But if it comes to *TP53*-EC association, high TP53 expression was observed by Kudela et al. [13] in poorly differentiated (G3) EC tumors. According to these Czech researchers, *TP53* may serve as one of the genetic markers responsible for differentiation of patients with bad EC prognosis [13]. Having found two meta-analyzes on *TP53* polymorphism-EC linkage, the authors of the first one did not observe any correlation between EC risk and *TP53* codon 72 polymorphism (although further studies in larger number of patients are recommended) [14], while the second report varies, combining Pro allele and Pro carriers (Pro/Pro, Arg/Pro) with increased EC risk (OR = 1.25 and 1.34, respectively) [15].

From the current point of view, it seems to be more important to analyze the studies focusing on the combined effect of *TP53* and *MDM2* polymorphisms in EC. Ashton et al. [16] reported that the combination of *MDM2* SNP309 and three *TP53* codon 72 polymorphisms is associated with a higher grade of EC (OR = 4,15). The Australian researchers found also the association between family history of breast cancer and *TP53* polymorphisms, suggesting a low-risk familial cancer grouping in this case (OR = 2.78) [16]. Nunobiki et al. calculated that homozygous *TP53* codon 72 Arg/Arg genotype together with homozygous *MDM2* SNP309 G/G polymorphism is responsible for significantly enhanced EC risk (OR = 3.28). The Japanese scientists concluded that these two polymorphisms may cooperatively increase the risk of EC among Japanese women [17]. Similar results were presented by Yoneda et al. [18] founding that the combination of *TP53* codon 72 Arg/Arg and *MDM2* SNP309 G/G+T/G significantly enhances the risk of EC (OR = 2.53).

Our results not only confirmed the fact that *MDM2* SNP309 G/G plus *TP53* codon 72 Arg/Arg genotype increases EC risk, but also showed that *MDM2* SNP309 T/T plus *TP53* codon 72 Pro/Arg genotype could decrease its risk. Additionally, some important connections with grading (GI–GIII) and staging (SI–SII) of EC were presented.

## Conclusions

The co-occurrence of selected gene polymorphisms of *MDM2* SNP309 and *TP53* codon 72 may influence the risk of EC, having also a relationship with grading and staging of this cancer.
